# Comparison of Two AI Chatbots for Diagnosis and Providing Treatment Suggestions in Retinopathy of Prematurity: Retrospective Study

**DOI:** 10.2196/86726

**Published:** 2026-10-07

**Authors:** Shaojuan Peng, Xinyu Zhao, Zhenquan Wu, Duo Yuan, Na Duan, Kaixuan Cui, Zhen Yu, Weihua Yang, Wenbin Wei, Wei Chi, Guoming Zhang

**Affiliations:** 1 Jinan University, Guangzhou, China; 2 Shenzhen Eye Medical Center, Shenzhen Eye Hospital, Southern Medical University, 18 Zetian Road, Futian District, Shenzhen, 518043, China, 1 0755-23959600; 3 Huizhou Third People’s Hospital, First Affiliated Hospital of Jinan University, Guangzhou, China; 4 Beijing Tongren Eye Center, Capital Medical University, Beijing, China

**Keywords:** Artificial intelligence chatbot, retinopathy of prematurity, wide-field fundus image, diagnosis, treatment suggestion

## Abstract

**Background:**

Retinopathy of Prematurity (ROP) is a leading cause of preventable childhood blindness; yet, a global shortage of experienced pediatric ophthalmologists impedes timely diagnosis and treatment. While emerging AI chatbots are promising clinical decision-support tools in some ophthalmic diseases, their performance in ROP diagnosis and providing treatment suggestions remains uncertain.

**Objective:**

This study aimed to compare the performance of Google’s Gemini 2.5 Pro and OpenAI’s ChatGPT o4-mini in ROP diagnosis and providing treatment suggestions against the gold standard of clinical consensus.

**Methods:**

A retrospective analysis was conducted on 70 infants (140 eyes) with treatment-requiring ROP, each providing structured clinical text data and wide-field fundus images. We adopted a 2-stage prompting strategy for AI chatbots, instructing them first to generate ROP diagnoses (including zone, stage, and presence of plus disease), and subsequently to provide treatment suggestions. After collecting the generated responses, we assessed their performance by comparing the consistency of their diagnosis and treatment suggestions with the consensus gold standard. Furthermore, 2 independent specialists quantitatively assessed the outputs of Gemini 2.5 Pro and ChatGPT o4-mini using the ROP-specific Global Quality Score (GQS), which is a 5-point scale ranging from 1 (unusable) to 5 (excellent). Statistical significance was set at *P*<.05, and all statistical analyses were performed using R (version 4.4.1; R Foundation for Statistical Computing).

**Results:**

For the tasks of ROP zoning and staging, the consistency rates between Gemini 2.5 Pro and ChatGPT o4-mini were 79.3% (111/140) vs 85.7% (120/140; zone), 64.3% (90/140) vs 70.0% (98/140; stage), respectively. For the task of treatment requirement, the rates (also referred to as sensitivity) were 93.6% (131/140) vs 90.7% (127/140), respectively. None of these differences were statistically significant (*P*>.05). However, Gemini 2.5 Pro showed significantly better performance in plus disease identification (consistency: 108/140, 77.1% vs 80/140, 57.1%; *P*=.006), while ChatGPT o4-mini demonstrated significantly higher guideline adherence in treatment modality suggestions based on gold-standard ROP diagnoses (consistency: 91/140, 65.0%; vs 55/140, 39.3%; *P*=.01). Compared to Gemini 2.5 Pro, ChatGPT o4-mini performed better in providing ROP treatment suggestions (GQS score; *P*=.001), while the 2 AI chatbots had comparable GQS scores in diagnostic tasks.

**Conclusions:**

ChatGPT o4-mini shows greater promise in generating evidence-based treatment suggestions based on gold-standard diagnoses, whereas Gemini 2.5 Pro shows advantages in visual interpretation, supporting its potential for targeted ROP diagnostic screening, particularly in identifying plus disease. As these AI chatbots continue to evolve, their performance merits further validation using larger cohorts.

## Introduction

Retinopathy of prematurity (ROP) is one of the leading causes of preventable childhood blindness worldwide, and its effective prevention and control have become an urgent global public health challenge [1,2]. Particularly in many middle-income countries [3], the improvement of neonatal intensive care capabilities has significantly increased the survival rate of low-birth-weight infants [2]. However, this increase has been accompanied by a rising incidence of ROP, which exacerbates the burden of childhood blindness [4]. Given the rapidly growing demand for ROP screening and treatment, the global shortage of experienced pediatric ophthalmologists has become increasingly severe, particularly in resource-limited regions [5,6]. In such regions, auxiliary diagnostic and treatment tools can reduce screening costs while supporting ophthalmologists in expanding service coverage through telemedicine [7,8]. This has further driven an unprecedented need for scalable, accurate, and standardized auxiliary tools [9].

The clinical management of ROP strictly adheres to evidence-based guidelines, and its diagnosis and treatment suggestions depend highly on the precise interpretation of fundus vascular and lesion features [10]. The third edition of the International Classification of ROP (ICROP3) updated and refined key indicators, including lesion zone, stage, and plus disease [11]. Wide-field fundus imaging is crucial not only for accurate diagnosis but also for providing objective visual evidence. These include the extent and morphology of the avascular retinal area, which are the core basis for guiding interventions such as laser therapy or anti–vascular endothelial growth factor (VEGF) injection [10,12]. In recent years, efficient feature preservation segmentation networks have further optimized the boundary delineation of fundus structures and lesions, thereby improving the reliability of AI-driven image interpretation [13]. To bridge the gap between these sophisticated diagnostic requirements and the global shortage of expert ophthalmologists, innovative auxiliary tools are urgently needed. Accordingly, AI chatbots that can integrate clinical text data and medical images may offer potential support in ROP diagnosis and providing treatment suggestions [14,15].

In recent years, AI chatbots such as OpenAI’s ChatGPT [16], Google’s Gemini [17], and DeepSeek (Liang Wenfeng) [18] have demonstrated tangible efficacy in diagnosis and providing treatment suggestions for ophthalmic diseases [19-21]. For fundus disorders such as retinal detachment, age-related macular degeneration, and macular hole, both ChatGPT and Google Gemini exhibited consistent assessments of clinical records, showing high concordance with retinal specialists’ evaluations [22,23]. Similarly, for other major fundus diseases, including age-related macular degeneration, glaucoma, and vitreous hemorrhage, ChatGPT showed preliminary diagnostic potential based solely on fundus photographs, although its performance remained variable [24]. The uncertain efficacy of AI chatbots in ROP diagnosis and providing treatment suggestions warrants a comparative analysis of their performance using a text-image fusion approach.

To compare the performance of Gemini 2.5 Pro and ChatGPT o4-mini in ROP diagnosis and providing treatment suggestions, we collected 70 infants with treatment-requiring ROP and evaluated the performance of the two AI chatbots. This study provides key empirical evidence and improvement directions for the application, evaluation, and iterative optimization of the 2 AI chatbots in pediatric ophthalmology and other imaging-driven clinical specialties.

## Methods

### Overview

This retrospective study was conducted at Shenzhen Eye Hospital (SZEH) and ultimately enrolled 70 infants (140 eyes in total) with ROP who underwent initial treatment for analysis in 2024. The entire process is illustrated in Figure 1.

**Figure 1.**
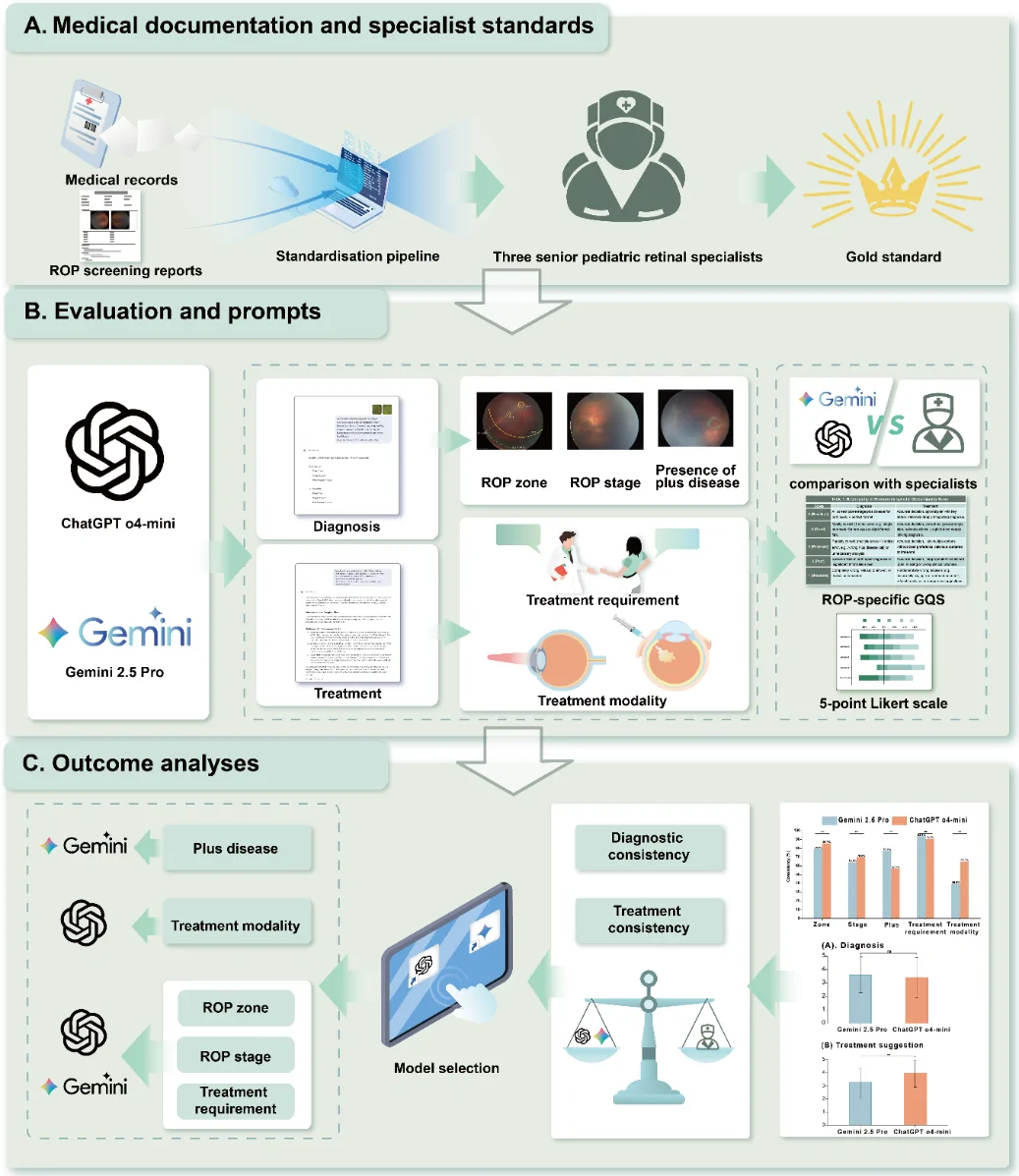
Study design and process for comparing the performance of two AI chatbots in the diagnosis and providing treatment suggestions for retinopathy of prematurity. The process consisted of 3 key steps: (A) medical documentation and specialist standards; (B) evaluation and prompts; (C) outcome analyses. GQS: Global Quality Score; ROP: retinopathy of prematurity.

### Medical Documentation and Specialist Standards

We collected demographic and clinical data for each infant using a standardized structured template, including sex, gestational age (GA), birth weight (BW), pregnancy type, and mode of delivery [25]. All structured fields were presented to both AI chatbots in an identical format to ensure fair comparisons. GA was recorded in weeks, and BW in grams, using a uniform numerical format. Clinical data comprised preoperative, deidentified bilateral fundus images obtained from ROP screening reports.

To ensure the reliability of the gold standard used for evaluating AI chatbot performance, we invited 3 senior pediatric retinal specialists (XZ, ZW, and GZ) to independently review the same ROP cases. Each specialist was provided with clinical text records and corresponding deidentified bilateral fundus images. Their tasks included (1) confirming the “gold standard” diagnosis for each case, which involved defining the ROP zone, stage, and presence of plus disease in accordance with the ICROP3 guidelines [11]; (2) establishing the optimal treatment suggestion, including the requirement for treatment and the selection of treatment modality (laser therapy or anti-VEGF injection). Fleiss kappa coefficient was used to assess interrater reliability. If all 3 specialists (XZ, ZW, and GZ) reached a unanimous agreement on the diagnosis and treatment, this consensus was finalized as the gold standard. Any disagreements were resolved through group discussion until a consensus was reached.

### Selection of Infants With ROP

Inclusion criteria were applied to ensure data integrity and quality: (1) preterm infants with GA <32 weeks or BW <2000 g, (2) ROP diagnosed according to the ICROP3 guidelines[11] and who underwent initial laser therapy or anti-VEGF injection, (3) complete clinical data available, including ROP screening reports (binocular ROP zone, stage, and plus disease assessment), preoperative wide-field fundus images, and treatment records.

Exclusion criteria were as follows: (1) incomplete medical history or clinical information, (2) absence of key preoperative or postoperative reports, (3) previous ROP treatment at other hospitals, (4) ROP stage ≥4 (due to more complex treatment regimens and limited feasibility of standardized surgical intervention).

### Evaluation and Prompt

#### Selection of AI Chatbots

The rapid expansion of AI chatbot research in health care [26-28] has witnessed the emergence of mainstream models such as OpenAI’s ChatGPT [16], Google’s Gemini [17], and DeepSeek [18], along with their subversions. Early versions such as ChatGPT-3.5 only supported text interaction and were unable to process medical images [29,30]. Among available Gemini subversions, we selected Gemini 2.5 Pro because complex ROP assessment requires detailed ophthalmic image interpretation and task-specific validation of multimodal reasoning in ophthalmology [31,32]. Specifically, Gemini 2.5 Pro supports text and image inputs, and provides a long context window, making it suitable for processing structured clinical records and fundus images in this text-image fusion task [33]. The development of medical vision-language models and foundation models in ophthalmology further supports the potential value of multimodal AI systems for ophthalmic image interpretation, while also highlighting the need for task-specific validation [34]. For ChatGPT o4-mini, the HealthBench framework released by OpenAI in 2025 provides a relevant reference for evaluating clinical reasoning and safety in medical AI systems [35,36]. OpenAI documentation indicates that ChatGPT o4-mini supports text and image inputs and is optimized for fast and effective reasoning, including visual tasks [37,38]. This design is aligned with the technical challenge of simultaneously interpreting clinical records and wide-field fundus images in ROP diagnosis and providing treatment suggestions [21].

Therefore, our study selected Gemini 2.5 Pro and ChatGPT o4-mini for evaluation, as these AI chatbots meet the rigorous requirements of clinical tasks for multimodal data integration. Gemini 2.5 Pro was publicly available through Google’s official platform during the study period [33]. ChatGPT o4-mini used in this study refers to the reasoning-focused o4-mini model released by OpenAI in April 2025 [37], distinct from the earlier ChatGPT-4o mini omni model released in 2024. We used its web-exclusive high-inference mode, an enhanced reasoning variant optimized for complex multimodal and clinical reasoning tasks. All chatbot interactions in this study were conducted exclusively via the official web interfaces of the respective providers, without reliance on API access. These standard web interfaces do not provide end-users with adjustable parameters such as temperature. The temperature value is fixed at a default setting determined by the platform providers and cannot be modified by users. Consequently, all AI chatbot outputs were generated under the default, fixed configuration of the web platforms to ensure consistency and reproducibility.

#### AI Chatbot-Aided Diagnosis and Treatment Suggestions

We conducted standardized AI chatbot interactions with Gemini 2.5 Pro and ChatGPT o4-mini between June 1 and June 30, 2025. To reduce evasive responses that AI chatbots may generate when directly asked for medical advice (eg, stating “I am not a medical professional and cannot provide treatment suggestions”) [22], this study adopted a 2-stage, specialist-simulation prompting strategy. The initial prompt framed the task as a “clinical simulation analysis scenario” and assigned the AI chatbots the role of an “experienced pediatric fundus disease specialist” with additional context: “This is a clinical simulation analysis scenario. You will act as a pediatric ophthalmologist with rich clinical experience, and based on the basic medical history and a varying number of Retcam fundus images provided, briefly analyze the ROP zone, stage, and whether there is plus disease in this infant (report separately for left and right eyes, omitting the analysis process and directly providing the results).”

In the diagnosis task, the 2 AI chatbots were provided with standardized clinical records and deidentified bilateral fundus images (consistent with those obtained by experts) for each infant. The left and right fundus images were uploaded as separate files (1600×1200 resolution) within this conversation, rather than as composite images with white dividing bars, thus minimizing potential interpretation errors related to image boundaries. Simultaneously, the two AI chatbots were asked: “As a pediatric ophthalmologist with rich clinical experience, based on the provided patient history and Retcam fundus images provided above, please directly report the ROP zone, stage, and presence of plus disease in this infant”

In the treatment suggestion task, we manually provided the gold-standard diagnoses (not the chatbot’s own outputs) to the AI chatbots, with the following instruction: “Based on my revised diagnoses of this infant, does the child require surgical intervention? If so, what is your recommended optimal surgical plan? Please briefly explain the rationale behind your suggestion”

For each infant, the diagnosis and treatment suggestion tasks were conducted within the same continuous chat session. For different infants, separate independent chat sessions were used with no information carryover between cases. A single query was submitted per case, and only the initial response was included for analysis. All model hyperparameters (eg, temperature and top-p sampling) were retained at the default values of the official web interface, with no custom parameter adjustments. Screenshots of the response interfaces of the two AI chatbots are shown in Figures 2 and 3. All prompts and interactions were conducted in Chinese and that the English prompts shown in the figures are translations. This design separated the AI chatbots’ performance in providing evidence-based treatment suggestions from diagnostic errors, enabling an unbiased evaluation based on the gold-standard diagnoses. At the end of each AI chatbot interaction, 2 specialists (XZ and ZW) independently graded the quality of the AI chatbots’ outputs using an ROP-specific Global Quality Score (GQS) adapted from Bernard et al [39].

**Figure 2.**
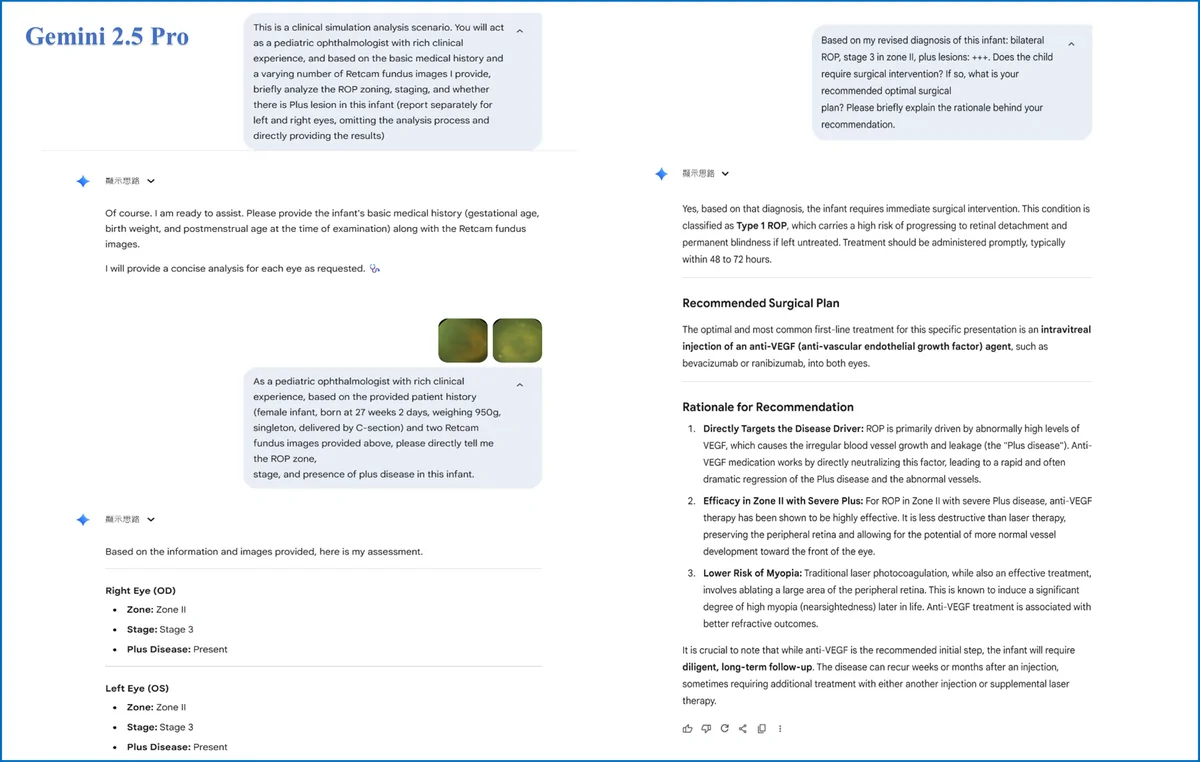
Screenshot of Gemini 2.5 Pro’s response interface for an infant with retinopathy of prematurity.

**Figure 3.**
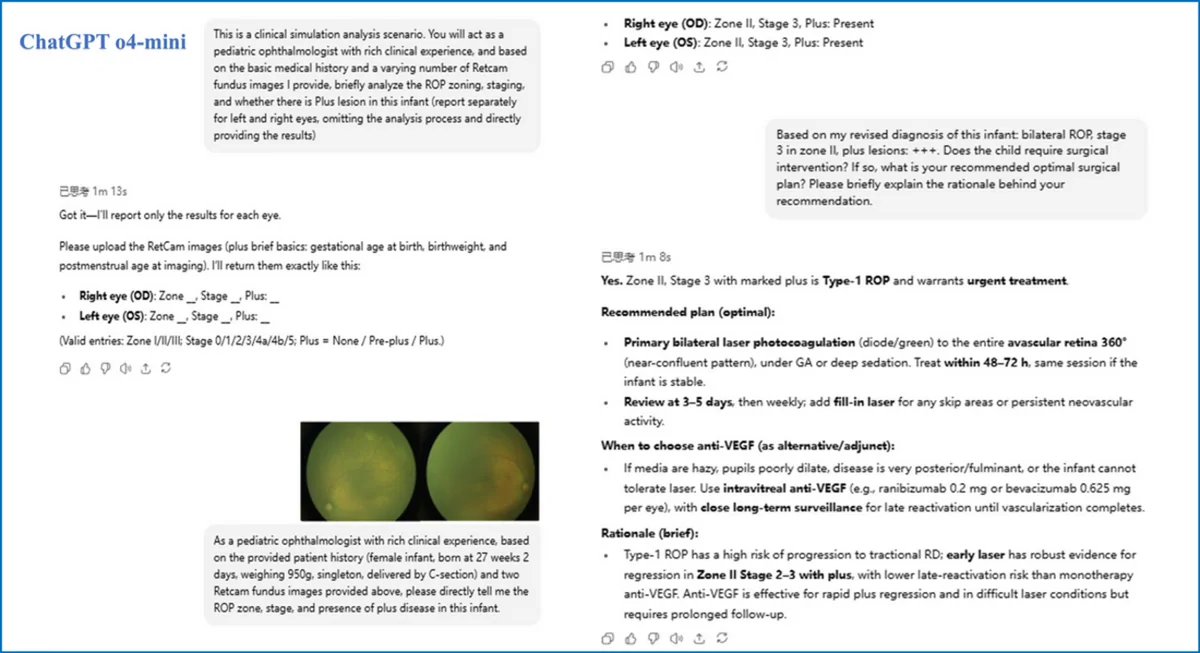
Screenshot of ChatGPT o4-mini’s response interface for an infant with retinopathy of prematurity.

The primary outcome of this study was to explore the performance of Gemini 2.5 Pro and ChatGPT o4-mini in analyzing infants with ROP and generating coherent, accurate diagnoses and treatment suggestions by comparing their outputs to the specialists’ consensus gold standard (including consistency rates of diagnoses and treatment suggestions). For treatment modality selection, a response that selects an effective treatment modality consistent with gold-standard decision criteria was rated as correct. As a secondary outcome, we used the mean GQS to quantify the subjective quality and clinical utility of the 2 AI chatbots’ output results.

#### ROP-Specific GQS

As shown in Table 1, the scoring system adopted a 5-point Likert scale (1=unusable, 5=excellent).

**Table 1.** Retinopathy of prematurity specific Global Quality Score criteria.

Score	Diagnosis	Treatment suggestion
5 (Excellent)	All bilateral diagnoses fully consistent with the gold standard	The treatment suggestions consistent with the gold standard, integrate individual characteristics and ICROP3^a^ guidelines, mention posttreatment monitoring, no hallucinations
4 (Good)	5 diagnoses consistent with the gold standard	The treatment suggestions consistent with the gold standard, integrate ICROP3 guidelines, lack of individualized details or posttreatment monitoring, no hallucinations
3 (Moderate)	3‐4 diagnoses consistent with the gold standard	The treatment suggestions inconsistent with the gold standard, integrate ICROP3 guidelines, no hallucinations
2 (Poor)	1‐2 diagnoses consistent with the gold standard	The treatment suggestions inconsistent with the gold standard, not integrate ICROP3 guidelines, hallucinations exist
1 (Unusable)	No diagnosis consistent with the gold standard or no response provided	The treatment suggestions contain fundamental errors (eg, no treatment required) or no suggestion is provided, hallucinations exist

a ICROP3: third edition of the International Classification of retinopathy of prematurity.

The ROP-specific GQS was adapted from the tool developed by Bernard et al [39], originally designed for website usability assessment. Given the lack of standardized quantitative instruments for evaluating clinical AI chatbots’ outputs, the GQS has been adopted in ophthalmic AI studies [40] and digital health research [41], supporting its use in our investigation. To better assess the quality of AI chatbot outputs in real ROP case analyses, we modified the GQS according to the ICROP3 guidelines [11] to generate ROP-specific GQS. We also incorporated hallucination assessment for treatment suggestion outputs [42]. Prior to scoring, 2 ROP specialist raters (XZ and ZW) underwent standardized training covering ICROP3 criteria and ROP-specific GQS scoring rules, with pilot calibration and discussion of borderline cases. To minimize bias, AI chatbots’ outputs were deidentified and provided as plain text, ensuring raters were blinded to the source of AI chatbots. Interrater reliability for assessing AI chatbots was evaluated using the intraclass correlation coefficient (ICC).

### Statistical Analysis

All statistical analyses were performed using R (version 4.4.1; R Foundation for Statistical Computing). The “gold standard” was established by the consensus of 3 pediatric retinal specialists (XZ, ZW, and GZ). Interrater reliability regarding ROP zone, stage, plus disease, treatment requirement, and treatment modality were assessed using the Fleiss kappa coefficient. Any disagreements among the 3 pediatric retinal specialists were resolved through discussion until a consensus was reached. Generalized estimating equations (GEE) were used to compare the consistency rates and GQS of the 2 models while accounting for intereye correlation. As all enrolled eyes in this study were treatment-requiring ROP, the metric for treatment requirement was equivalent to sensitivity. Subgroup analyses of the 2 AI chatbots for diagnosis and providing treatment suggestions were conducted between Zone I and Zone II (as shown in Table 2), because Zone III cases were insufficient for statistical analysis. For the 3 Zone III eyes, both ChatGPT o4-mini and Gemini 2.5 Pro misclassified all 3 eyes as Zone II. For the Zone I subgroup, complete separation was observed for Gemini 2.5 Pro in both the treatment requirement and treatment modality comparisons, as it achieved 100% correct classification. This separation precludes the use of conventional GEE Wald-type estimates due to their unreliability. Consequently, for these 2 comparisons, we applied a penalized GEE approach incorporating Firth likelihood penalty, which provides reliable estimates of the regression coefficients while properly accounting for the inherent correlation between eyes. For the GQS, each AI chatbot had 140 individual rater scores for each task. The mean GQS for each response was calculated by averaging the scores from the two raters, followed by the overall mean score. Score category frequencies and hallucination rates were calculated using the original integer scores provided by both raters. *P*<.05 was considered statistically significant.

**Table 2.** Subgroup analysis of two AI chatbots for diagnosis and providing treatment suggestions in Zones I and II.

Outcome	Zone I^a^ (n=15)				Zone II^b^ (n=122)			
Gemini 2.5 Pro, n (%)	ChatGPT o4-mini, n (%)	OR^c^ (95% CI)	*P* value	Gemini 2.5 Pro, n (%)	ChatGPT o4-mini, n (%)	OR (95% CI)	*P* value
Zone identification	10 (66.7)	2 (13.3)	0.08 (0.01-0.63)	.02	100 (82.0)	118 (96.7)	6.49 (1.53-27.55)	.01
Stage	9 (60.0)	12 (80.0)	2.70 (0.69-10.64)	.16	80 (65.6)	84 (68.9)	1.16 (0.60-2.23)	.66
Plus disease	13 (86.7)	13 (86.7)	1.00 (0.04-26.23)	1.0	94 (77.0)	65 (53.3)	0.34 (0.17-0.68)	.003
Treatment requirement	15 (100)	14 (93.3)	0.18^d^ (0.02-1.54)	.12^d^	113 (92.6)	110 (90.2)	0.73 (0.45-1.17)	.19
Treatment modality	15 (100)	5 (33.3)	0.01^d^ (0.002-0.05)	<.001^d^	40 (32.8)	83 (68.0)	4.36 (1.75-10.88)	.002

a For Zone I, no significant differences were observed in most outcomes, with the exception of ROP zone identification and treatment modality.

b For Zone II, ChatGPT o4-mini significantly outperformed Gemini 2.5 Pro in ROP zone identification (OR 6.49; *P*=.011) and treatment modality selection (OR 4.36; *P*=.002). Conversely, ChatGPT o4-mini showed significantly lower consistency in plus disease identification compared with Gemini 2.5 Pro (OR 0.34; *P*=.003). *P*<.05 was considered statistically significant.

c OR: odds ratio.

d For the Zone I subgroup, complete separation occurred for Gemini 2.5 Pro in the treatment requirement and treatment modality comparisons (100% correct). Accordingly, a Firth-penalized GEE was applied to address this separation. Other statistical comparisons were performed using GEE. All ORs reflect ChatGPT o4-mini versus Gemini 2.5 Pro.

### Ethical Considerations

The study was approved by the Institutional Review Board (IRB) of SZEH (IRB No. 2025KYPJ120) prior to its initiation. Informed consent was waived by the IRB due to the retrospective study design and the use of fully deidentified data for secondary analysis. To ensure compliance with ethical standards and data privacy regulations, all sensitive personal information was removed from case descriptions, only ROP-related key factors such as GA and BW were retained to balance research accuracy and privacy protection. All fundus images were anonymized, free of any potential biometric identifiers or embedded textual metadata, and subjected to quality control in adherence to data privacy protocols. This study did not recruit any additional subjects and no compensation was provided to participants.

## Results

### Overview

Demographic characteristics of the 70 infants are shown in Table 3. Specifically, the mean GA was 26.79 (SD 2.14) weeks, and the mean BW was 888.60 (SD 277.82) g. Among all infants, 57.14% (40/70) were male; 64.29% (45/70) were singleton pregnancies; and the proportion of vaginal deliveries was comparable to that of cesarean sections.

**Table 3.** Demographic characteristics of infants with retinopathy of prematurity (N=70).

Characteristics	Values
Sex, n (%)	
Male	40 (57.14)
Female	30 (42.86)
Gestational age (weeks), mean (SD)	26.79 (2.14)
Birth weight (g), mean (SD)	888.60 (277.82)
Pregnancy type, n (%)	
Singleton	45 (64.29)
Multiple pregnancy	25 (35.71)
Delivery type, n (%)	
Vaginal delivery	37 (52.86)
Cesarean section	33 (47.14)

Diagnosis and treatment suggestions for the 140 eyes (70 infants) are shown in Table 4. The most common diagnosis was Zone II, Stage 3 ROP. Specifically, Zone II involvement was observed in 62 of 70 left eyes (88.6%) and 60 of 70 right eyes (85.7%). Stage 3 was noted in 63 of 70 left eyes (90.0%) and 64 of 70 right eyes (91.4%). Furthermore, plus disease was highly prevalent, affecting 64 of 70 eyes (91.4%) bilaterally. All eyes required surgical treatment. Laser therapy was the primary modality, administered to 50 of 70 left eyes (71.4%) and 48 of 70 right eyes (68.6%). The remaining eyes were treated with anti-VEGF injections.

**Table 4.** Baseline characteristics of infants with retinopathy of prematurity (ROP; N=70).

Characteristic and category		Left eye, n (%)	Right eye, n (%)
ROP Zone			
Zone I		7 (10.0)	8 (11.4)
Zone II		62 (88.6)	60 (85.7)
Zone III		1 (1.4)	2 (2.9)
ROP Stage			
Stage 1		1 (1.4)	1 (1.4)
Stage 2		6 (8.6)	5 (7.1)
Stage 3		63 (90.0)	64 (91.4)
Plus disease			
Absent (-)		6 (8.6)	6 (8.6)
Present (+)		64 (91.4)	64 (91.4)
Treatment requirement			
Yes		70 (100.0)	70 (100.0)
Treatment modality			
Laser therapy		50 (71.4)	48 (68.6)
IVI^a^		20 (28.6)	22 (31.4)

a IVI: intravitreal injection.

### Interrater Reliability

Interrater reliability among the 3 specialists for the gold standard was substantial to excellent across all 5 tasks: Fleiss kappa for ROP zone=0.65 (95% CI 0.36‐0.82), for ROP stage=0.72 (95% CI 0.43‐0.90), for plus disease=0.66 (95% CI 0.34‐0.86), and for treatment modality=0.82 (95% CI 0.68‐0.92). For treatment requirement, all 3 specialists (XZ, ZW, and GZ) reached unanimous consensus, resulting in no calculated kappa value for this endpoint. Initial agreement was achieved in 84.3% (59/70) of cases; the remaining 11 cases with disagreement were resolved through discussion until full consensus was reached for all tasks. Subsequently, the consistency of GQS scoring for two AI chatbots’ outputs was evaluated by two independent ROP specialists (XZ and ZW) raters to ensure reliability of the secondary outcome measures. For Gemini 2.5 Pro, excellent interrater reliability was observed for both diagnostic GQS consistency (ICC=0.85; 95% CI 0.77‐0.90) and treatment suggestion GQS consistency (ICC=0.81; 95% CI 0.71‐0.88). Similarly, ChatGPT o4-mini demonstrated strong interrater reliability for diagnostic GQS consistency (ICC=0.86; 95% CI 0.78‐0.91) and treatment suggestion GQS consistency (ICC=0.83; 95% CI 0.66‐0.91).

### Overall Performance Comparison of Two AI Chatbots

We compared the consistency between Gemini 2.5 Pro and ChatGPT o4-mini in 5 core clinical tasks (ROP zone, stage, plus disease identification, treatment requirement, and treatment modality). As shown in Figure 4, Gemini 2.5 Pro and ChatGPT o4-mini demonstrated comparable consistency in ROP zoning (79.3% vs 85.7%), staging (64.3% vs 70.0%), and treatment requirement (93.6% vs 90.7%), with no statistically significant differences (all *P*>.05). However, Gemini 2.5 Pro demonstrated significantly better consistency in diagnosing plus disease (77.1% vs 57.1%; *P*=.006), suggesting its advantage in visual pattern recognition tasks. In contrast, ChatGPT o4-mini showed significantly better consistency in treatment modality (65.0% vs 39.3%; *P*=.01), indicating strengths in providing treatment suggestions based on the gold standard diagnoses. As shown in Table 2, subgroup analyses revealed distinct strengths and limitations between the two AI chatbots across ROP zones. All odds ratios (ORs) reflect ChatGPT o4-mini versus Gemini 2.5 Pro. In Zone I, Gemini 2.5 Pro maintained superior consistency, particularly in treatment modality task (100.0% vs 33.3%; OR=0.01; *P*<.001). Conversely, in Zone II, ChatGPT o4-mini significantly outperformed Gemini 2.5 Pro in ROP zone identification (96.7% vs 82.0%; OR=6.49; *P*=.01) and treatment modality selection (68.0% vs 32.8%; OR=4.36; *P*=.002). In addition, ChatGPT o4-mini showed significantly lower consistency in plus disease detection within Zone II compared with Gemini 2.5 Pro (53.3% vs 77.0%; OR=0.34; *P*=.003).

**Figure 4.**
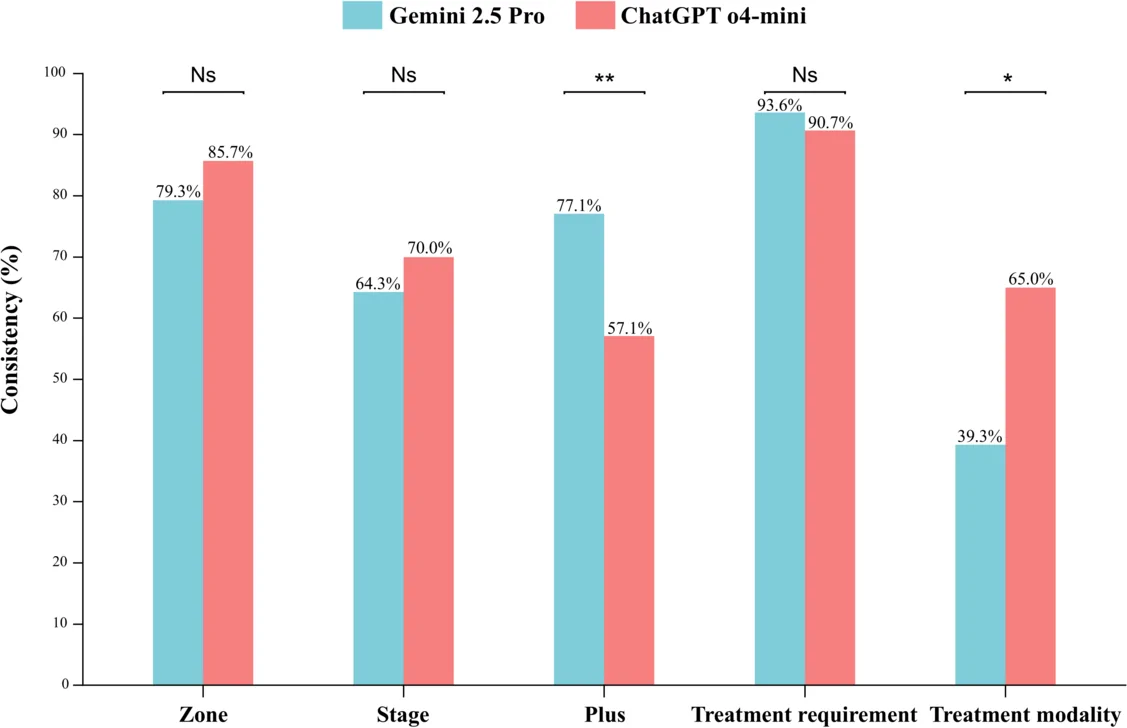
Comparison of consistency between Gemini 2.5 Pro and ChatGPT o4-mini in diagnosis and providing treatment suggestions for retinopathy of prematurity. The Y-axis represents consistency (%). The X-axis denotes specific tasks (ROP zoning, staging, plus disease identification, treatment requirement, treatment modality). Ns: not significant; *:P<.05, significant difference; **: *P*<.01, significant difference. Gemini 2.5 Pro outperformed ChatGPT o4-mini in plus disease identification (*P*=.006), whereas ChatGPT o4-mini was superior in treatment modality selection (*P*=.01).

### Subgroup Analyses of Two AI Chatbots According to ROP Zone

We conducted subgroup analyses to explore the two AI chatbots’ performance across distinct ROP risk zones, including Zone I (high-risk) and Zone II (intermediate-risk), to identify potential strengths and limitations within specific populations.

As shown in Table 2, in Zone I (n=15), ChatGPT o4-mini achieved numerically lower consistency rates than Gemini 2.5 Pro in zone identification (13.3% vs 66.7%; OR=0.08; *P*=.02), and treatment modality (33.3% vs 100%; OR=0.01; *P*<.001). No significant differences were observed for ROP staging (*P*=.16), plus disease identification (*P*>.99), and treatment requirement (*P*=.12) in Zone I.

In Zone II (n=122), ChatGPT o4-mini exhibited clear advantages in clinical decision-making. It significantly outperformed Gemini 2.5 Pro in both ROP zone identification (96.7% vs 82.0%; OR=6.49; *P*=.01) and treatment modality selection (68.0% vs 32.8%; OR=4.36; *P*=.002). Conversely, ChatGPT o4-mini showed significantly lower consistency in plus disease identification compared with Gemini 2.5 Pro (53.3% vs 77.0%; OR=0.34; *P*=.003). For ROP staging and treatment requirement, no statistically significant differences were observed between the two AI chatbots in Zone II.

### GQS Results Analysis

The GQS results are presented in Figure 5. Based on the content evaluated by the GQS, hallucinations in treatment suggestion were identified in ratings of 1 or 2. For the diagnosis task, Gemini 2.5 Pro obtained a higher mean GQS score (3.61; SD 1.42) than ChatGPT o4-mini (3.41; SD 1.45), with no statistically significant difference (*P*=.39). Notably, the similar SDs across both AI chatbots suggest comparable variability in diagnostic output quality. Gemini 2.5 Pro had score 1 ratings in 7.14% (10/140) and score 2 ratings in 12.14% (17/140), while ChatGPT o4-mini yielded score 1 ratings in 7.86% (11/140) and score 2 ratings in 15.71% (22/140). In terms of treatment suggestion task, ChatGPT o4-mini significantly outperformed Gemini 2.5 Pro (mean GQS: 3.97; SD 1.26 vs 3.29; SD 1.19; *P*=.001). The small difference in SDs suggests similar variability in treatment suggestion output quality. Gemini 2.5 Pro had score 1 ratings in 7.86% (11/140) and score 2 ratings in 14.29% (20/140), corresponding to a rating-level hallucination rate of 22.14% (31/140), while ChatGPT o4-mini yielded score 1 ratings in 7.14% (10/140) and score 2 ratings in 7.14% (10/140), corresponding to a rating-level hallucination rate of 14.29% (20/140). Hallucinations may manifest as proposing ineffective treatment modalities not endorsed by the ICROP3 guidelines, exaggerating the indications for a particular intervention, or fabricating unproven reasons for a treatment modality preference. These results showed that ChatGPT o4-mini achieved a significantly higher mean GQS score than Gemini 2.5 Pro for treatment suggestions and received fewer hallucination-related ratings, supporting its superior performance in providing clinical ROP treatment suggestions in this study.

**Figure 5.**
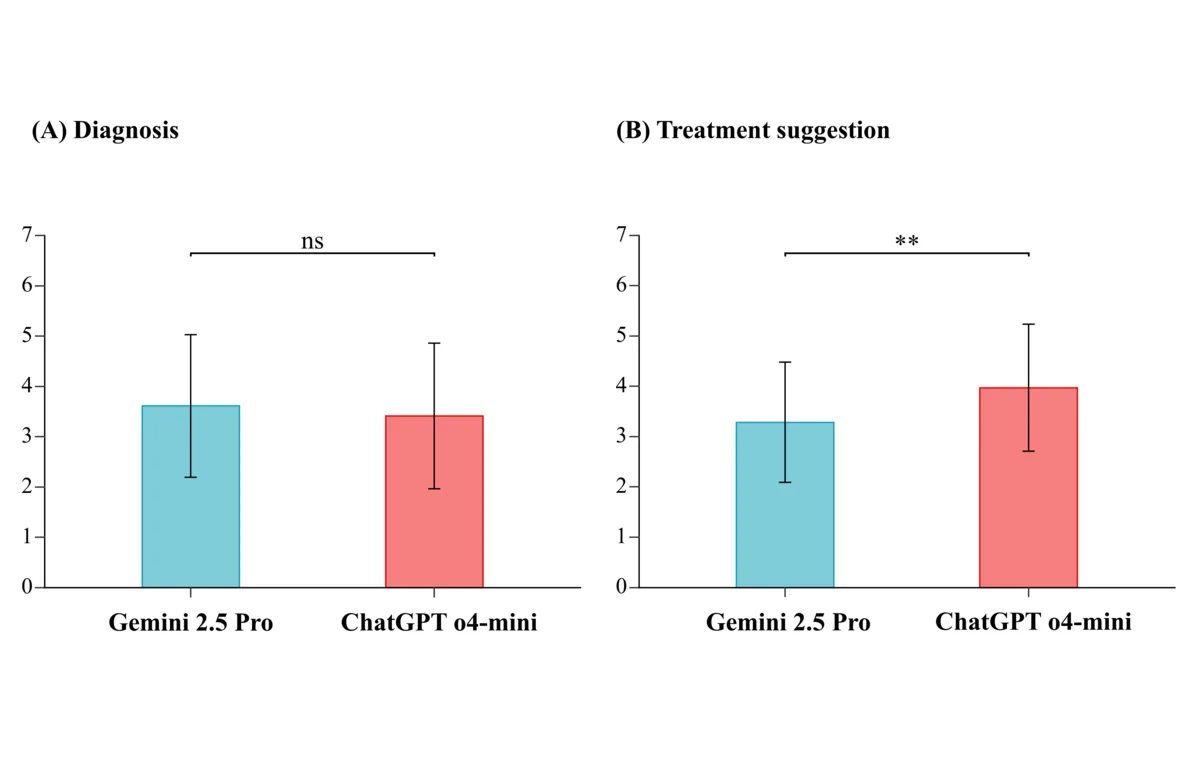
Comparison of global quality scores (GQS) for diagnosis and providing treatment suggestion in retinopathy of prematurity between Gemini 2.5 Pro and ChatGPT o4-mini. The bar chart displays the mean GQS for both (A) Diagnosis and (B) Treatment suggestion. For diagnostic task, the average GQS was 3.61 for Gemini 2.5 Pro vs 3.41 for ChatGPT o4-mini (*P*=.39). For treatment suggestion task, the average GQS was 3.29 for Gemini 2.5 Pro vs 3.97 for ChatGPT o4-mini (*P*=.001). Error bars represent SD. ns: not statistically significant difference; **(*P*<.01 (significant difference).

## Discussion

### Principal Findings and Comparison to Prior Work

This study compared the performance of Gemini 2.5 Pro and ChatGPT o4-mini for diagnosing and providing treatment suggestions in ROP. The results provided key empirical evidence for the performance of AI chatbots to bridge the gap between the complex diagnostic requirements of ROP and the shortage of pediatric ophthalmologists. Furthermore, our findings may offer directions for improvement, with potential implications for the wider application and evaluation of AI chatbots in other imaging-driven specialties, consistent with the broader translational framework of AI in smart health care [43].

The tasks of ROP zoning, staging, and treatment requirement are relatively structured because they are based on standardized disease classification and treatment decision criteria provided by the ICROP3 guidelines [11]. This guideline helps AI chatbots transform complex clinical observation tasks into highly standardized and repeatable operational procedures, thus demonstrating consistently reliable performance in these tasks [11,44]. Both AI chatbots were provided with the gold-standard diagnoses (zone, stage, and presence of plus disease) before answering the question “Is surgical treatment required?,” as this task was highly objective and did not require complex clinical reasoning. Based on these standardized features, the 2 AI chatbots in this study showed relatively consistent performance on these tasks by leveraging their multimodal processing capabilities and the standardized criteria provided by the ICROP3 guidelines [11,45].

The identification of plus disease in ROP requires precise visual discrimination of morphological changes in retinal vasculature [46]. Gemini 2.5 Pro exhibited superior consistency in identifying plus disease in this study. This advantage may be related to the broader ability of modern vision-language models to integrate visual features through multimodal representation learning and adaptive feature integration mechanisms [47,48]. In contrast, although ChatGPT o4-mini supports text-image reasoning and visual tasks according to OpenAI documentation [49], its lower consistency in plus disease identification in this study suggests that general-purpose multimodal reasoning may not fully translate into optimal recognition of subtle ROP vascular abnormalities. This performance difference appears to be task-dependent. Prior ophthalmology studies have shown that the relative performance of AI chatbots varies across examination-based and clinical ophthalmology tasks [31,50,51]. This finding does not contradict our results but rather highlights the “performance specialization” of AI chatbots [50,51]. These differences may be associated with model-specific optimization strategies, training data composition, input format, and task types [45,52]. Specifically, the training data varied: our study used wide-field fundus images, whereas the exam study used typical test-related images. Furthermore, these tasks impose different cognitive demands: our study focused on high-fidelity visual discrimination, whereas exam-based studies require textual integration of visual materials of diverse complexity. Therefore, vision-optimized AI chatbots such as Gemini 2.5 Pro are more adaptable for clinical tasks that rely on fine visual pattern recognition, as their performance aligns closely with the visual dependency of the task.

In tasks of determining treatment modality, which demand the integration of multidimensional clinical information (including clinical text records and wide-field fundus images), AI chatbots need to simulate specialists’ multifactor trade-off logic. Consistent with prior ophthalmic multimodal AI chatbot research [21], ChatGPT o4-mini may have benefited from integrating structured text records and fundus images when deducing the optimal treatment modality, yielding superior performance. Previous medical large language model studies also suggest that language models can perform well on structured medical knowledge and reasoning tasks [53,54]. For questions based on established medical knowledge with clear and objective answers, these AI chatbots achieved strong performance. This indicates that when tasks are governed by explicit rules and objective criteria, the performance gap among different AI chatbot architectures narrows, and their capabilities converge.

Subgroup analysis showed that in high-risk Zone I, characterized by severe posterior retinal lesions and vascular abnormalities [11], Gemini 2.5 Pro outperformed ChatGPT o4-mini in ROP zone identification and treatment modality selection. This finding may again reflect Gemini 2.5 Pro’s stronger task-specific visual discrimination in cases requiring fine vascular pattern recognition, suggesting its potential value in the identification of complex retinal vascular diseases. Conversely, in the intermediate-risk Zone II with relatively mild vascular lesions, ChatGPT o4-mini significantly outperformed Gemini 2.5 Pro in both zone identification and treatment modality selection. Since treatment decisions in Zone II rely more heavily on the comprehensive clinical context rather than isolated visual examination results [11], ChatGPT o4-mini may have benefited from integrating structured clinical records and fundus images when generating treatment suggestions, consistent with prior ophthalmic multimodal AI chatbot research [21]. It is worth noting that ChatGPT o4-mini’s ability to identify plus disease is not as good as Gemini 2.5 Pro, which is consistent with the overall comparison results. The above results confirm that the performance of the AI chatbot is closely related to the risk of the ROP zone. Gemini 2.5 Pro is more suitable for high-risk Zone I cases that require precise visual identification, while ChatGPT o4-mini is more suitable for medium-risk Zone II cases. This also verifies the overall performance of the two AI chatbots in different clinical scenarios and their robustness in handling different retinal complexities.

The difference in GQS between ChatGPT o4-mini and Gemini 2.5 Pro varies across task dimensions, reflecting the interplay between specialist scoring logic and AI chatbot performance. According to our ROP-specific GQS criteria, specialists mainly evaluated completeness and clinical practicality. When scoring, specialists particularly focus on whether the AI chatbots’ output fully covers the core diagnostic elements of ROP cases and whether it conforms to the follow-up or treatment suggestions. In the diagnosis dimension, no significant difference in GQS was observed between the two AI chatbots, indicating comparable performance in capturing ROP diagnostic details. In contrast, ChatGPT o4-mini achieved a significantly higher GQS in the treatment suggestion dimension. This advantage may be partly explained by its structured output format, which is compatible with clinical workflows and may help specialists extract key information efficiently [55]. However, because the training data and optimization details of commercial AI chatbots are not fully disclosed, this interpretation should be regarded as a task-specific observation rather than a confirmed model-level mechanism. Although Gemini 2.5 Pro performs well in isolated image analysis, it often experiences logical discontinuities when integrating multidimensional textual information of ROP cases, resulting in a decreased matching degree between the output and actual clinical requirements.

Notably, the SD patterns differed between the 2 AI chatbots. For Gemini 2.5 Pro, the SDs for diagnostic GQS were higher than that for treatment suggestion GQS, while ChatGPT o4-mini exhibited higher SDs in treatment suggestion GQS. This aligns with the common observation of insufficient output consistency in AI chatbots for complex clinical decisions, particularly evident in multimodal information integration tasks [56]. The output quality and stability of AI chatbots are positively associated with their ability to structure clinical knowledge and are influenced by task complexity, further supporting the emphasis on transparency, usability, and validity in explainable AI systems for clinical decision support [57].

### Limitations

This study has several limitations. First, we evaluated AI chatbots performance on moderately severe and well-documented ROP cases (excluding stage 4 and above) rather than the full clinical spectrum encountered in routine ROP screening scenarios, failing to reflect real-world data quality challenges and limiting the clinical applicability of our findings. Furthermore, the single-center convenience sampling design and potential selection bias restrict geographic and demographic generalizability. Notably, although some findings from the Zone I subgroup analysis reached statistical significance, these results should be interpreted with caution due to the small sample size, and validation in larger cohorts is still needed. Second, given the exploratory nature of this study, formal adjustments for multiple comparisons (eg, Bonferroni correction) were not applied to avoid an inflated risk of Type II error (false-negative conclusions). While this represents a potential limitation, the robust effect sizes observed for the primary endpoints largely mitigated this concern. Third, the lack of cross-platform validation across different software and hardware environments limits the generalizability of the AI chatbots and their reliability in diverse clinical settings. Fourth, the AI chatbots have different training data cutoffs, resulting in asynchronous knowledge updates that may be inconsistent with contemporary clinical practice standards, with Gemini 2.5 Pro trained up to January 2025 and ChatGPT o4-mini up to June 2024. This discrepancy in training data cutoff dates between the two AI chatbots may also contribute to the observed differences in their diagnostic performance. Fifth, there is still a potential model hierarchy mismatch between Gemini 2.5 Pro (flagship high-computation model) and ChatGPT o4-mini (efficiency-focused model), despite our use of the enhanced reasoning mode of ChatGPT o4-mini. Sixth, our prompt instructions forced the 2 AI chatbots to omit analytical processes and directly output results, which may have artificially lowered their performance. The prompts did not explicitly request eye-specific separate treatment suggestions, though the models spontaneously generated treatment suggestions for each eye separately, which may affect the accuracy of our analysis results. Furthermore, in the treatment suggestion task, we manually input gold-standard diagnoses rather than using the chatbots’ own outputs, and all diagnostic and therapeutic queries were completed within a single continuous chat session, which precluded evaluation of their end-to-end clinical management performance. All interactions were continuous, so the treatment suggestions generated by chatbots were not produced within an isolated conversational context. Moreover, all interactions were conducted in Chinese, and the AI chatbot performance may vary across prompting languages. Finally, the absence of standardized tools to quantify “hallucinations in treatment suggestion” prevents objective assessment of potential risks in chatbot outputs, weakening their credibility as clinical decision support tools.

### Future Directions

While our results, validated against specialist consensus as the gold standard, appeared promising, rigorous external validation—including multicenter studies, prospective trials, and cross-platform verification—is essential before clinical implementation to confirm generalizability and ensure patient safety. Future studies can validate the end-to-end clinical management performance of AI chatbots based on their own independent diagnoses, rather than manual input of gold-standard diagnoses. In addition, further balanced cross-model comparisons are also needed to explore how model size, parameter count, and architecture shape the performance of AI chatbots in multimodal ROP tasks.

### Conclusion

In this study of ROP, ChatGPT o4-mini appears to be more promising for evidence-based treatment suggestions based on the gold standard diagnoses, while Gemini 2.5 Pro’s visual acuity highlights its potential for targeted ROP diagnostic screening, particularly in identifying plus disease. As these AI chatbots continue to evolve, further validation in larger and more diverse cohorts is warranted to establish their clinical utility and generalizability.

## Abbreviations

BW: birth weight
GA: gestational age
GEE: generalized estimating equation
GQS: Global Quality Score
ICC: intraclass correlation coefficient
ICROP3: the third edition of the International Classification of ROP
IRB: Institutional Review Board
OR: odds ratio
ROP: retinopathy of prematurity
SZEH: Shenzhen Eye Hospital
VEGF: vascular endothelial growth factor
